# Determinants of *Aedes* mosquito density as an indicator of arbovirus transmission risk in three sites affected by co-circulation of globally spreading arboviruses in Colombia, Ecuador and Argentina

**DOI:** 10.1186/s13071-021-04984-z

**Published:** 2021-09-19

**Authors:** Benoit Talbot, Beate Sander, Varsovia Cevallos, Camila González, Denisse Benítez, Claudio Carissimo, María C. Carrasquilla Ferro, Neris Gauto, Sergio Litwiñiuk, Karen López, Mario I. Ortiz, Patricio Ponce, Stephany D. Villota, Fabian Zelaya, Mauricio Espinel, Jianhong Wu, Marcos Miretti, Manisha A. Kulkarni

**Affiliations:** 1 School of Epidemiology and Public Health, University of Ottawa, Ottawa, ON Canada; 2 Dalla Lana School of Public Health, University of Toronto, Toronto, ON Canada; 3 Toronto General Hospital Research Institute, University Health Network, Toronto, ON Canada; 4 Development and Innovation Management Unit (CZ9), Instituto Nacional de Investigación en Salud Pública, Quito, Ecuador; 5 Center for Investigation in Tropical Microbiology and Parasitology, Universidad de los Andes, Bogotá, Colombia; 6 Municipal Institute for Surveillance and Control of Vectors, Municipalidad de Posadas, Posadas, Argentina; 7 Group for Investigation in Applied Genetics (GIGA), IBS, UNaM-CONICET, Posadas, Argentina; 8 School of Medicine, Universidad Laica Eloy Alfaro de Manabí, Manta, Ecuador; 9 Department of Mathematics and Statistics, York University, Toronto, ON Canada

**Keywords:** Disease risk, Global health, Mosquito vector, Socio-economic status, Wealth Index

## Abstract

**Background:**

The global impact of Zika virus in Latin America has drawn renewed attention to circulating mosquito-borne viruses in this region, such as dengue and chikungunya. Our objective was to assess socio-ecological factors associated with *Aedes* mosquito vector density as a measure of arbovirus transmission risk in three cities of potentially recent Zika virus introduction: Ibagué, Colombia; Manta, Ecuador; and Posadas, Argentina, in order to inform disease mitigation strategies.

**Methods:**

We sampled *Aedes* mosquito populations in a total of 1086 households, using indoor and peridomestic mosquito collection methods, including light traps, resting traps, traps equipped with chemical attractant and aspirators. For each sampled household, we collected socio-economic data using structured questionnaires and data on microenvironmental conditions using iButton data loggers.

**Results:**

A total of 3230 female *Aedes* mosquitoes were collected, of which 99.8% were *Aedes aegypti* and 0.2% were *Aedes albopictus*. Mean female *Aedes* mosquito density per household was 1.71 (standard deviation: 2.84). We used mixed-effects generalized linear Poisson regression analyses to identify predictors of *Aedes* density, using month, neighborhood and country as random-effects variables. Across study sites, the number of household occupants [incidence rate ratio (IRR): 1.08, 95% confidence interval (CI): 1.01–1.14], presence of entry points for mosquitoes into the household (IRR: 1.51, 95% CI: 1.30–1.76) and presence of decorative vegetation (IRR: 1.52, 95% CI: 1.22–1.88) were associated with higher *Aedes* density; while being in the highest wealth tertile of household wealth (IRR: 0.78, 95% CI: 0.66–0.92), knowledge of how arboviruses are transmitted (IRR: 0.94, 95% CI: 0.89–1.00) and regular emptying of water containers by occupants (IRR: 0.79, 95% CI: 0.67–0.92) were associated with lower *Aedes* density.

**Conclusions:**

Our study addresses the complexities of arbovirus vectors of global significance at the interface between human and mosquito populations. Our results point to several predictors of *Aedes* mosquito vector density in countries with co-circulation of multiple *Aedes*-borne viruses, and point to modifiable risk factors that may be useful for disease prevention and control.

**Graphical Abstract:**

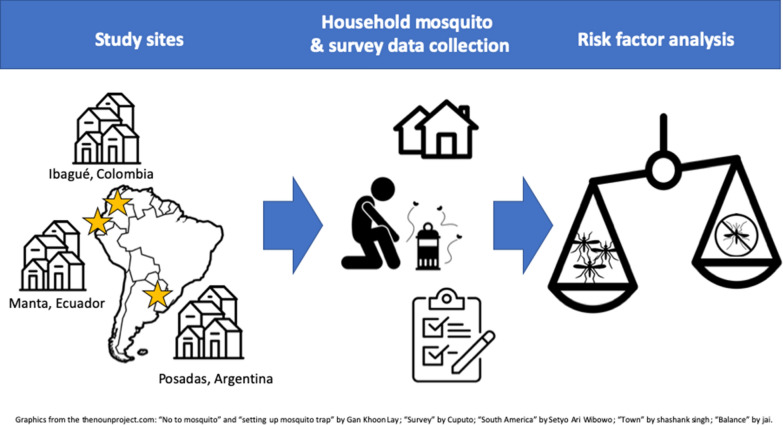

**Supplementary Information:**

The online version contains supplementary material available at 10.1186/s13071-021-04984-z.

## Background

Globalization and global environmental changes have increased the exposure of world populations to a number of emerging pathogens, including tropical mosquito-borne arboviruses [1, 2]. Among globally emerging and re-emerging arboviruses causing human disease in recent years, Zika, dengue and chikungunya viruses have been of increasing regional importance in the Americas. Zika virus was historically confined to Africa and Asia [3]. In late 2014 this virus spread from the South Pacific Islands and French Polynesia and reached the Americas [4], where it was associated with severe autoimmune and neurological complications, such as Guillain-Barré syndrome and microcephaly in newborn infants [5]. Dengue virus is the cause of the most common arboviral disease that affects humans, dengue fever, which may lead to death in severe hemorrhagic fever cases [6]. Chikungunya virus is associated with long-lasting arthralgia, rash and fever, and has caused outbreaks of disease in countries in Africa, Asia and Europe [7]. In 2013, this virus emerged in the Americas in Saint-Martin, and rapidly spread throughout the Latin America and Caribbean region within a year [7].

Two mosquito species, *Aedes aegypti* and *Aedes albopictus*, are among the world’s most prominent arboviral vectors. The original geographic range of *Ae. aegypti* in sub-Saharan Africa was extended to the Americas and the Asia–Pacific region, when the species was introduced via ships during the European colonization period [8]. This mosquito species has since become domesticated (i.e. adapted to breed in human habitats) and is now established across all of these regions. The original geographic range of *Ae. albopictus* in Asia has expanded through global trade particularly since the 1980s [9], and this mosquito species is now found across nearly half of the entire world landmass [10–12]. *Aedes aegypti* and *Ae. albopictus* are able to survive and establish reproducing populations if thresholds in temperature, humidity and precipitation are met [13, 14]. Their close association to human habitations and human host specificity means they display high vector competence, infection rates and transmission rates for Zika, dengue and chikungunya viruses in the Americas [15].

The impact of urbanization and social equity on mosquito vector density and disease risk has been the subject of a large body of literature (e.g. [16–18]). While lower socio-economic status (SES) and poverty seem to be strongly associated with disease risk in general [19–21], and more specifically with mosquito-borne disease risk [18, 22], the actual relationship between *Aedes* mosquito density and socio-economic profile is unclear. A variety of studies exploring this subject suggest that *Aedes* density is either associated with higher SES, lower SES or not associated at all with the socio-economic profile of neighborhoods, instead possibly depending on a wide range of contributing or confounding factors, including the geographic scale of the investigation [23]. In addition to differences in study location and/or study design, the discrepancy between these results could lie in the distinction between household-level wealth and neighborhood-level socio-economic profile (e.g. [22]). Neighborhood SES has been associated with factors such as public water and waste management [24] and population density [25], while household-level SES has been associated with the quality of household structure (e.g. presence of window screens) [22], education [26] and specific mosquito- and arbovirus-related knowledge [27], with higher SES generally associated with lower *Aedes* density. However, the presence and type of indoor and outdoor vegetation, including potted plants that are often found in and around households with higher SES, are also associated with *Aedes* breeding sites [28]. The complex relationship between household wealth and other household characteristics on *Aedes* mosquito density needs to be investigated further in areas displaying geographic and temporal variations in meteorological and climatic factors in order to better understand the main determinants of *Aedes* vector density and, by extension, arboviral disease risk.

The Latin American region is diverse given its range of different meteorological and climatic conditions, shaped by its geography and topography, but also owing to the tremendous socio-economic inequality and variation within and among political boundaries. The impact of climatic variation on *Aedes* density has been demonstrated in numerous studies, such as those investigating the contribution of temperature, rainfall and humidity [13, 14, 29, 30] and seasonality [31, 32]. The contribution of socio-economic variation to *Aedes* density has also been demonstrated in numerous studies in the region, most notably in Mexico, Colombia and Brazil [23]. While the socio-economic profile was associated with *Aedes* density in most studies, the direction of the effect was mixed.

In the present study, our main objective was to investigate the proximal determinants of *Aedes* mosquito density at the household level, including factors related to household characteristics, microenvironment, crowding, water and waste management, mosquito protection behavior and arbovirus knowledge by household occupants, to identify entry points for intervention. Based on previous literature, we expected that household characteristics and household occupant characteristics and behaviors would affect *Aedes* mosquito density in the household. We conducted the study in three cities within the Latin American region that reflect variations in meteorological, climatic and socio-economic conditions, and differences in incidence/prevalence of arboviral diseases.

## Methods

### Study sites and neighborhood selection

We selected three study sites to represent different eco-epidemiological settings in the Latin American region: the cities of Ibagué, Colombia; Manta, Ecuador; and Posadas, Argentina. Ibagué is a city with approximately 500,000 inhabitants, situated in a valley of the mountainous region of the northern Andes, at an altitude of 1285 m a.s.l., and displays a tropical rainforest climate with consistent rain and warm weather throughout the year. Manta is a Pacific coastal city with approximately 5000 inhabitants, situated at 6 m a.s.l., which displays a tropical arid climate, with hot weather and little rainfall throughout the year. Posadas is a city with approximately 400,000 inhabitants, situated on the shore of the Paraná river, at an altitude of 120 m a.s.l., and displays a humid subtropical climate, which varies seasonally between a hot and humid summer and a warm winter. For each study site, we defined two classes of neighborhood SES, namely high and low, based on government classification or available information, including population density, household crowding, household wealth and services available. Given these measures vary between study site and are specific to the metrics that were applied in the respective countries, we mainly used these approximations as a way to ensure we selected neighborhoods displaying a representative level of socio-economic variation. We then selected two neighborhoods representative of each class, where the implementation of household surveys and mosquito collection activities were possible in at least 96 households. Each month over a period of 12 months, in each of the four neighborhoods of each of the three study sites, we randomly sampled eight households without replacement for the collection of household survey data and mosquito sampling, for a target sample size of 384 households per study site, or 1152 households across the three sites.

### Mosquito collection and identification

For each sampled household, we collected information on date of sampling and latitude and longitude of the household. We placed an iButton (Maxim Integrated Inc., San Jose, CA, USA) in a central location inside the household in the morning, for collection of average temperature and humidity data over a 24-h period. We searched for any potential mosquito breeding sites inside and around the household and noted their presence and type. We subsequently identified the peridomestic and intradomestic spaces of the household for mosquito sampling. The peridomestic space was defined as the outdoor areas immediately surrounding the household, and could include indoor spaces where occupants did not typically spend most of their time, such as garages, laundry rooms, etc., while the intradomestic space was defined as the indoor area where occupants spent most of the time for sleeping, cooking, eating and other daily activities. For each household, in the peridomestic space we placed one CDC miniature light trap, which uses a light as attractant, and one CDC resting trap, which uses a textured surface as attractant (Centre for Disease Control and Prevention, Atlanta, GA, USA), and in the intradomestic space, we placed one BG-Sentinel2 trap equipped with a BG lure, which is used as a chemical attractant (Biogents Inc., Cambridge, MA, USA). We set all traps to collect mosquitoes for a 24-h period. On the following day, we used a Prokopack aspirator (John W. Hock Company, Gainesville, FL, USA) to actively collect mosquitoes in both the intradomestic and peridomestic spaces, for 30 min. Therefore, a similar sampling effort was applied to each household. Finally, we brought collected mosquito samples from traps and aspirators to a local field laboratory, and we identified all *Aedes* spp. individuals to the species, using standard taxonomic keys [33]. Identified specimens were then stored in RNAlater (Thermo Fisher Scientific, Waltham, MA, USA) and transported to a laboratory for subsequent processing and molecular analysis for virus detection; the results of these analyses are to be presented in separate papers (e.g. [34]).

### Household questionnaire survey, arbovirus knowledge, wealth index and socio-economic profile

For each sampled household, we administered a survey questionnaire to the household respondent (see Additional file 1: Household questionnaire survey). The questionnaire contained categories of questions pertaining to general characteristics of the household, water availability, waste disposal, mosquito control, demographics and arbovirus knowledge. Households with missing survey responses for more than 50% of all questions were removed from the dataset as the survey questionnaire was deemed to be incomplete. We calculated an arbovirus knowledge score using answers from questions RK1, RK5, RK9 (i.e. knowledge about existence of Zika, dengue and chikungunya viruses, respectively), RK13a (i.e. knowledge about the main mode of transmission of the three viruses, which is through mosquito feeding), RK14b and RK14c (i.e. knowledge about the two main protection strategies against these viruses, which is reduction of breeding sites and protection against mosquitoes, respectively) (see Additional file 1: Household questionnaire survey). For each of these questions, a positive answer is directly related to the level of general knowledge of the respondent on the risks associated with mosquitoes in and around their household. Therefore, we summed answers for these questions, up to a maximum of six, for each household of the study, which we denoted as the arbovirus knowledge predictor. We calculated a household-level wealth index using the methods of Vyas and Kumaranayake [35]. We first computed a principal components analysis, using the ‘stats’ package in R 3.6.0 (https://www.R-project.org/), based on household assets and whether the household had electricity service, using questions HC2 and HC5 (see Additional file 1: Household questionnaire survey), for all sampled households of the study. We then applied rotated weights from the first principal component to respective questionnaire variables and summed values for all variables at each household. We categorized data into tertiles to obtain three levels of household wealth across the entire dataset, which we denoted as the wealth index predictor. Neighborhoods included in the study were initially categorized as either perceived high or low socio-economic profile, based on country-specific definitions that varied across study sites. To compute a more objective measure of neighborhood socio-economic profile that is standardized across study sites, we calculated the proportion of households of the lowest wealth index tertile in each neighborhood.

### Analyses of household and occupant characteristics and behaviors

To attain our main objective, we investigated the effect of a wide range of factors related to characteristics of the household and its occupants on female *Aedes* mosquito density. These factors were carefully chosen a priori to include variables most likely affecting *Aedes* mosquito density, including aspects related to household occupant characteristics; household occupants’ mosquito protection behaviors; water and waste management behaviors; and indoor and outdoor household components and characteristics. These included average temperature, average humidity, altitude, arbovirus knowledge, wealth index and socio-economic profile, in addition to variables related to household construction, water supply and storage, waste management and breeding sites, among others (for the complete list of predictors considered, see Additional file 2: Table S1 and Additional file 3: Table S2). We conducted a mixed-effects simple generalized linear regression, using the ‘lme4’ package [36] in R 3.6.0, for each of the 53 predictor variables against captured household female *Aedes* mosquito density, using a hierarchical random-effects term of month of sampling, nested in sampling neighborhood, nested in study site. We used this random-effects structure due to the hierarchical nature of these terms, where temporal variation of mosquito density across months is specific to each study neighborhood, and spatial variation of mosquito density across neighborhoods is specific to each country. Prior to these analyses, we subtracted the mean and divided the product by the standard deviation of all values for numerical variables (see Additional file 2: Table S1). We successively performed these analyses using datasets specific to each study site, and using the entire dataset incorporating all study sites. For all analyses, we used the ‘na.exclude’ argument for the ‘na.action’ parameter to allow the prediction and residual functions to be run on the whole dataset [37].

We selected all predictors displaying a significant association (*P* < 0.05) with *Aedes* mosquito density in simple regression analyses, where no more than 95% of data points had the same value and no more than 75% of data were missing, to reduce issues caused by small cell size, and used these in a mixed-effects multiple generalized linear regression analysis, using the ‘lme4’ package [36] in R 3.6.0. Again, we used a hierarchical random-effects term of month of sampling, nested in sampling neighborhood, nested in study site. To assess the presence of multicollinearity in our multivariable regression models, we computed the generalized variance inflation factor GVIF^(1/(2×*Df*))^, using the ‘car’ package [38] in R 3.6.0, for all predictors. We then iteratively dropped the predictor associated with the highest GVIF^(1/(2×*Df*))^ value from the full model and reran the new regression model until all GVIF^(1/(2×*Df*))^ values were < 2. We then proceeded with a two-step model selection approach using the Bayesian information criterion (BIC), i.e. the Akaike information criterion (AIC) using the logarithm of the number of observations as the *k* parameter. We first reduced the dataset to remove all data points containing missing data at any of the predictors included in the full model, which is a condition for the approach when applied on mixed-effects regression models. Using the reduced dataset, we computed a BIC value for the full model and for all combinations of the full model excluding one predictor, using the ‘lme4’ package [36] in R 3.6.0. A BIC value decrease of 2 to 6 is considered positive evidence for a predictor displaying little effect on the response variable [39]. We ran a new multivariable regression model, but this time excluding all predictors that, when dropped from the full model, caused a decrease of the BIC value of > 5. We then reapplied the same model selection approach as a second step on this new model, but dropping predictors causing a decrease of the BIC value of > 4. Using this two-step model selection approach, we ensured that only the most important predictors were retained in the resulting final model. We ran the final model using the full dataset, and computed the incidence rate ratio (IRR) values (i.e. the exponents of the slope coefficients), the 95% confidence intervals for the IRR values and *P* values for each predictor. Finally, we computed the marginal and conditional* R*^2^ of the final model, using the ‘MuMIn’ package [40] in R 3.6.0. We successively performed these analyses using datasets specific to each study site, and using the entire dataset incorporating all study sites.

## Results

### Study sites and neighborhood selection

We sampled households in 16 neighborhoods, rather than the expected 12, across the three study sites in Ibagué, Colombia; Manta, Ecuador; and Posadas, Argentina. This higher number of neighborhoods was due to the lower average number of households in neighborhoods of perceived high SES in Ibagué, Colombia, and Manta, Ecuador, which led us to sample households in five and three neighborhoods of perceived high SES in these study sites, respectively. We did not obtain sufficient survey data in 66 of the 1152 target households, and we therefore removed these from the dataset due to incomplete responses. Our analyses therefore used data from a total of 1086 households: 379 in Ibagué, Colombia; 335 in Manta, Ecuador; and 372 in Posadas, Argentina. We sampled households over a total of 12 months per study site; however, the start date of data collection differed among study sites owing to site-specific logistic constraints and availability of mosquito trap supplies: June 2018 to May 2019 in Ibagué, Colombia; March 2019 to February 2020 in Manta, Ecuador; and January to December 2019 in Posadas, Argentina.

### Mosquito collection and identification

**Fig. 1:**
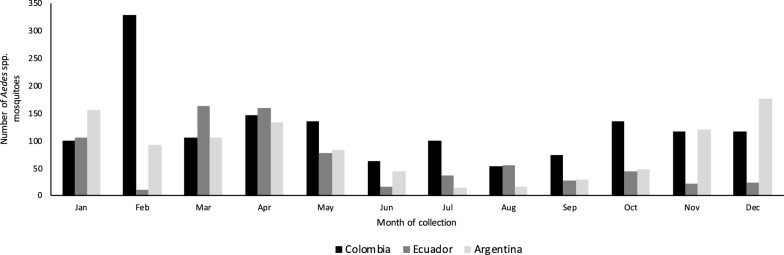
Number of captured mosquitoes identified as *Aedes* spp. by month of sampling (three-letter abbreviations) and study site in Ibagué, Colombia; Manta, Ecuador; and Posadas, Argentina

**Fig. 2:**
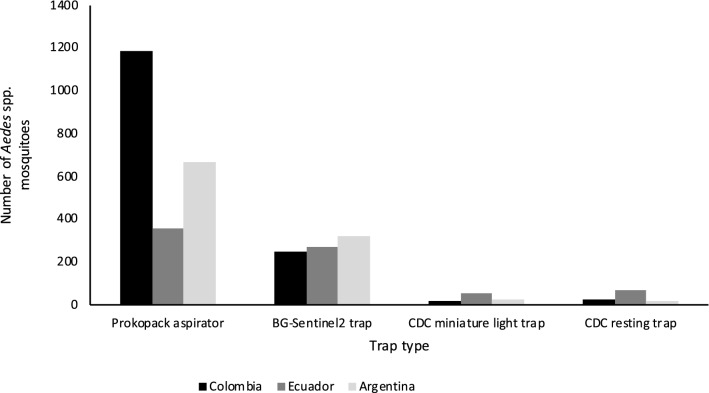
Number of captured mosquitoes identified as *Aedes* spp. by method of capture and study site in Ibagué, Colombia; Manta, Ecuador; and Posadas, Argentina

We collected a total of 3230 *Aedes* spp. mosquitoes belonging to two species, with 1470 collected from Ibagué, Colombia; 740 from Manta, Ecuador; and 1020 from Posadas, Argentina (Table 1). We collected seven *Ae. albopictus* individuals, all in Ibagué, while all 3223 remaining individuals were *Ae. aegypti*. The number of collected mosquitoes varied across months of collection, with June to September yielding smaller numbers across sites, and March to May yielding larger numbers across sites; between October and February the number varied considerably across sites (Fig. 1). The Prokopack aspirators yielded the largest number of collected mosquitoes at each site, BG-Sentinel2 traps yielded a few hundred captured mosquitoes at each site and CDC miniature light traps and CDC resting traps each yielded < 100 captured mosquitoes at each site (Fig. 2). Female *Aedes* spp. accounted for a little more than half of all collected *Aedes* spp. individuals, in all study sites (Table 1). The number of female *Aedes* spp. in any household varied between 0 and 31, with the average number of female *Aedes* spp. per household being 1.13 in Manta, Ecuador; 1.80 in Posadas, Argentina; and 2.12 in Ibagué, Colombia; the overall average was 1.71 (Table 1).

**Table.1:** Total number of sampled households, and total number, number of females and average female per household of captured mosquitoes identified as *Aedes* spp. by study site

Study site	Number of sampled households	Total number of *Aedes* spp.	Total number of female *Aedes* spp.	Range in number of female *Aedes* spp. per household	Average (SD) number of female *Aedes* spp. per household
Ibagué, Colombia	379	1470	805	0–9	2.12 (3.00)
Manta, Ecuador	335	740	381	0–21	1.13 (2.28)
Posadas, Argentina	372	1020	695	0–31	1.80 (3.05)
Total	1086	3230	1881	0–31	1.71 (2.84)

*SD* Standard deviation

### Household questionnaire survey, arbovirus knowledge, wealth index and socio-economic profile

**Fig. 3:**
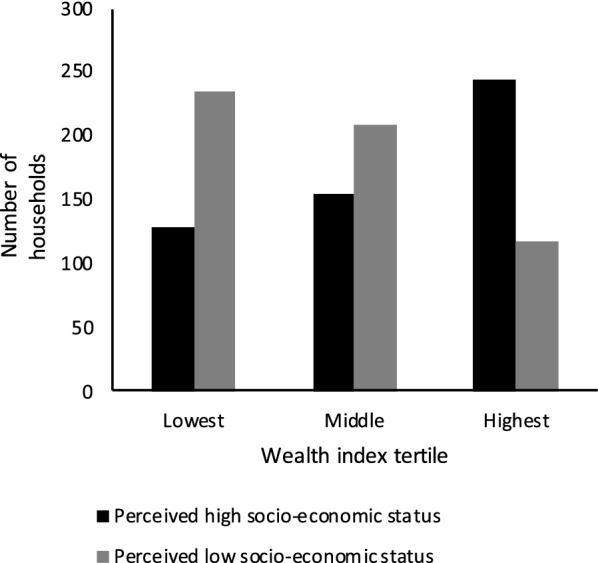
Number of households included in the study by wealth index tertile and whether they are located in a neighborhood that was perceived high or low socio-economic status prior to the study

**Fig. 4:**
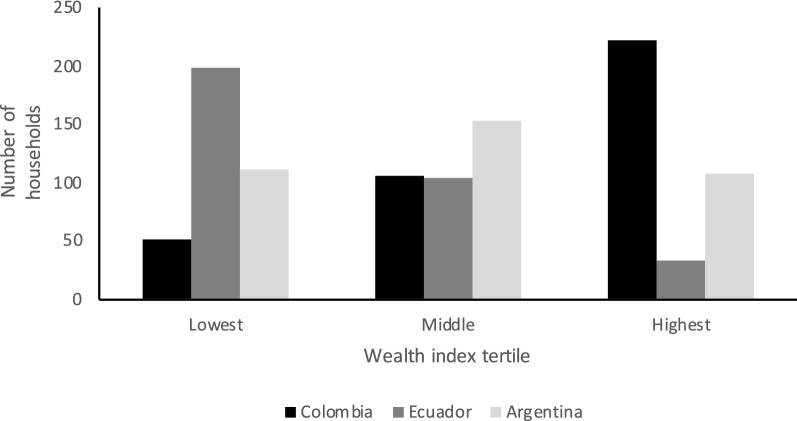
Number of households included in the study by wealth index tertile and study site in Ibagué, Colombia; Manta, Ecuador; and Posadas, Argentina

Average and standard deviation (SD) values and proportion of missing data for all numerical variables are shown in Additional file 2: Table S1. The proportion of values for each factor level and proportion of missing data for all categorical variables are shown in Additional file 3: Table S2. Upon close visual inspection for detection of systematic patterns in the missingness of data, we deemed that missing data were most likely to be caused by sporadic technical problems with the use and/or function of electronic equipment, such as the iButton data loggers and the tablets used to collect questionnaire data, and that, therefore, missingness pattern is completely random and therefore unlikely to impede our analyses [41]. Water management and household characteristics directly affecting mosquito population abundance varied across the three wealth index tertiles, with greater availability and use of mosquito control measures generally observed in households belonging to the highest wealth tertile (Table 2). Across study sites, arbovirus knowledge was 4.76 out of 6.00 (SD: 1.00). Households in the lowest and middle wealth index tertiles were mostly found in neighborhoods that were considered of low SES prior to the study, while households in the highest wealth tertile were mostly found in neighborhoods considered of high SES (Fig. 3). However, the reverse was not uncommon, with many households in the highest wealth tertile found in neighborhoods of perceived low SES, and many households in the lowest and middle wealth tertiles found in neighborhoods of perceived high SES. The majority of households in the lowest wealth tertile were in Manta, Ecuador, whereas the majority of households in the highest wealth tertile were in Ibagué, Colombia (Fig. 4).

**Table.2:** Number of households included in the study by wealth index tertile and the reported answer for a selection of 12 most biologically relevant questions from the household questionnaire survey

Wealth Index tertile:	Lowest			Middle			Highest		
Questionnaire answer^a^:	No	Yes	Ø	No	Yes	Ø	No	Yes	Ø
Biologically relevant question									
Running water as main source for drinking	134 (37)	227 (63)	1 (0)	108 (30)	254 (70)	0 (0)	84 (23)	278 (77)	0 (0)
Problem obtaining water	229 (63)	131 (36)	2 (1)	266 (73)	94 (26)	2 (1)	298 (82)	63 (17)	1 (0)
Storing water	274 (76)	86 (24)	2 (1)	219 (60)	143 (40)	0 (0)	166 (46)	196 (54)	0 (0)
Presence of large water container	210 (58)	115 (32)	37 (10)	174 (48)	116 (32)	72 (20)	119 (33)	185 (51)	58 (16)
Emptying containers	256 (71)	105 (29)	1 (0)	230 (64)	132 (36)	0 (0)	156 (43)	206 (57)	0 (0)
Washing containers	253 (70)	108 (30)	1 (0)	215 (59)	147 (41)	0 (0)	140 (39)	222 (61)	0 (0)
Presence of vegetation in pots	259 (72)	86 (24)	17 (5)	236 (65)	120 (33)	6 (2)	263 (73)	98 (27)	1 (0)
Using window screens	320 (88)	41 (11)	1 (0)	330 (91)	32 (9)	0 (0)	281 (78)	81 (22)	0 (0)
Presence of points of entry for mosquitoes	219 (60)	137 (38)	6 (2)	277 (77)	85 (23)	0 (0)	290 (80)	69 (19)	3 (1)
Permanent floor materials	6 (2)	354 (98)	2 (1)	2 (1)	360 (99)	0 (0)	0 (0)	362 (100)	0 (0)
Permanent roof materials	21 (6)	338 (93)	3 (1)	9 (2)	353 (98)	0 (0)	2 (1)	359 (99)	1 (0)
Permanent wall materials	14 (4)	346 (96)	2 (1)	3 (1)	359 (99)	0 (0)	2 (1)	359 (99)	1 (0)
Presence of breeding sites	255 (70)	60 (17)	47 (13)	271 (75)	60 (17)	31 (9)	291 (80)	53 (15)	18 (5)

Values are presented as the number of households with the percentage in each wealth index tertile given in parentheses (*n* = 362)

^a^Ø: missing data

### Analyses of household and occupant characteristics and behaviors

All results of simple regression analyses can be found in Additional file 2: Table S1 and Additional file 3: Table S2. Using the entire dataset across study sites, the final model contained 11 predictors, nine of which had an IRR that was significantly different from 1 (Table 3). Number of occupants in the household, number of years living in the household and using bed nets had an IRR value of between 1.05 and 1.35, and presence of points of entry into the household for mosquitoes and presence of other/decorative vegetation had a large IRR of > 1.50. Arbovirus knowledge, the highest wealth index tertile, emptying containers and presence of herbs had an IRR ranging from 0.75 to 0.95 (Table 3). Marginal and conditional* R*^2^ of the final model were 0.1 and 0.6, respectively.

**Table 3:** Mixed-effects regression incidence rate ratio (IRR) values, the two boundaries of the 95% confidence interval (IRR 2.5% and 97.5% CI) and the *P* value, for each predictor included in the final model of household female *Aedes* mosquito density for the entire dataset across study sites

Predictor^a^	Level^a^	IRR	IRR 2.5% CI	IRR 97.5% CI	*P*
Number of occupants		1.08	1.01	1.14	0.012*
Number of occupants who study		1.01	0.95	1.08	0.660
Years spent living in household		1.20	1.11	1.29	< 0.001*
Arbovirus knowledge		0.94	0.89	1.00	0.042*
Wealth index	Lowest	Reference			
	Middle	0.89	0.76	1.05	0.149
	Highest	0.78	0.66	0.92	0.004*
Using bed net	No	Reference			
	Yes	1.31	1.07	1.59	0.007*
Emptying containers	No	Reference			
	Yes	0.79	0.67	0.92	0.003*
Presence of points of entry for mosquitoes	No	Reference			
	Yes	1.51	1.30	1.76	< 0.001*
Presence of green areas around house	No	Reference			
	Yes	1.10	0.96	1.26	0.152
Presence of herbs	No	Reference			
	Yes	0.86	0.75	0.99	0.034*
Presence of other/decorative vegetation	No	Reference			
	Yes	1.52	1.22	1.88	< 0.001*

Estimation of model coefficients was conducted on a total of 741 households (68% of the full dataset). Average proportion of variance explained by random-effects factors, i.e. month, neighborhood and country was 0.51, 0.19 and 0.00, respectively

^a^Predictors that are underlined have an IRR that is significantly different from 1

*Significant at a threshold α = 0.05

Using the dataset from Ibagué, Colombia, our final model contained 14 predictors, 13 of which had an IRR that was significantly different from 1 (Table 4). Number of total occupants and number of students, number of floors in the household and presence of green areas and absence of water bodies near the household all had an IRR ranging from 1.10 to 1.50. Presence of other/decorative vegetation had a large IRR of 1.59, and waste collection methods, whether in a location outside the household or collection by municipal/private agency compared to inside the property, both had an exceptionally large IRR of > 8.00. Number of family cores, distance to nearest household and arbovirus knowledge, the highest wealth index tertile, using insecticide and other means of protection against mosquitoes (mostly electric rackets, fans and insecticide tablets) had an IRR between 0.65 and 0.90 (Table 4). Marginal and conditional* R*^2^ of the final model were 0.2 and 0.7, respectively.

**Table 4:** Mixed-effects regression IRR values, the two boundaries of the 95% CI (IRR 2.5% and 97.5% CI) and the *P* value, for each predictor included in the final model of household female *Aedes* mosquito density for the dataset from Ibagué, Colombia

Predictor^a^	Level^a^	IRR	IRR 2.5% CI	IRR 97.5% CI	*P*
Number of occupants		1.16	1.05	1.28	0.002*
Number of occupants who study		1.13	1.02	1.25	0.017*
Number of floors		1.23	1.11	1.34	< 0.001*
Number of family cores		0.78	0.70	0.87	< 0.001*
Distance to nearest household		0.89	0.80	0.98	0.019*
Arbovirus knowledge		0.87	0.81	0.94	< 0.001*
Wealth index	Lowest	Reference			
	Middle	0.87	0.69	1.11	0.257
	Highest	0.65	0.51	0.84	0.001*
Insecticide use	No	Reference			
	Yes	0.83	0.71	0.98	0.023*
Killing insects	No	Reference			
	Yes	0.82	0.62	1.06	0.129
Use of other means of protection against mosquitoes	No	Reference			
	Yes	0.68	0.53	0.85	0.001*
Presence of green areas around household	No	Reference			
	Yes	1.26	1.04	1.52	0.018*
Presence of water bodies near household	Yes	Reference			
	No	1.47	1.08	1.97	0.011*
Presence of other/decorative vegetation	No	Reference			
	Yes	1.59	1.26	2.00	< 0.001*
Waste collection method	Inside property	Reference			
	Outside property	9.63	2.03	176.58	0.026*
	Private or municipal collection	8.91	1.93	163.28	0.030*

Estimation of model coefficients was conducted on a total of 370 households (98% of the full dataset). Average proportion of variance explained by random-effects factors, i.e. month and neighborhood, was 0.23 and 0.84, respectively

^a^Predictors that are underlined have an IRR that is significantly different from 1

*Significant at a threshold α = 0.05

Using the dataset from Manta, Ecuador, our final model contained 11 predictors, five of which had an IRR that was significantly different from 1 (Table 5). Humidity had an IRR value of 1.39, and problem obtaining water and using insecticide had a large IRR > 2.00. Presence of container in the yard had an IRR value of 0.69, and the ‘weekly’ level of frequency of garbage collection had a small IRR value of 0.36 (Table 5). Marginal and conditional* R*^2^ of the final model were 0.3 and 0.5, respectively.

**Table 5:** Mixed-effects regression IRR values, the two boundaries of the 95% CI (IRR 2.5% and 97.5% CI) and the *P* value, for each predictor included in the final model of household female *Aedes* mosquito density for the dataset from Manta, Ecuador

Predictor^a^	Level^a^	IRR	IRR 2.5% CI	IRR 97.5% CI	*P*
Humidity		1.39	1.10	1.76	0.005*
Arbovirus knowledge		1.11	0.89	1.39	0.364
Presence of container in the yard	No	Reference			
	Yes	0.69	0.50	0.96	0.025*
Presence of water tank	No	Reference			
	Yes	1.04	0.73	1.47	0.839
Problem obtaining water	No	Reference			
	Yes	2.07	1.41	3.05	< 0.001*
Store water to wash	No	Reference			
	Yes	1.44	0.91	2.26	0.115
Store water to cook	No	Reference			
	Yes	0.95	0.68	1.31	0.733
Insecticide use	No	Reference			
	Yes	2.05	1.47	2.85	< 0.001*
Washing containers	No	Reference			
	Yes	1.11	0.53	2.29	0.782
Killing insects	No	Reference			
	Yes	0.79	0.45	1.35	0.386
Frequency of garbage collection	Every other day	Reference			
	Daily	1.59	0.98	2.62	0.064
	Unpredictable	0.95	0.40	2.09	0.894
	Weekly	0.36	0.12	0.92	0.041*

Estimation of model coefficients was conducted on a total of 176 households (53% of the full dataset). Average proportion of variance explained by random-effects factors, i.e. month and neighborhood, was 0.26 and 0.02, respectively

^a^Predictors that are underlined have an IRR that is significantly different from 1

*Significant at a threshold α = 0.05

Using the dataset from Posadas, Argentina, our final model contained 15 predictors, six of which had an IRR that was significantly different from 1 (Table 6). Number of family cores had an IRR value of 1.31, and the ‘unpredictable’ level of frequency of obtaining water, storing water and presence of points of entry for mosquitoes into the household had a large IRR of ≥ 1.70. The ‘property’ level of type of lease and using other means of protection against mosquitoes (mostly using ventilators and insecticide tablets) had a small IRR value of < 0.55 (Table 6). Marginal and conditional *R*^2^ of the final model were 0.4 and 0.5, respectively. Unfortunately, due to a large amount of missing data specifically in the dataset from Posadas, Argentina, only 92 households (25% of this study site’s dataset) could be used to compute model coefficients.

**Table 6:** Mixed-effects regression IRR values, the two boundaries of the 95% CI (IRR 2.5% and 97.5% CI) and the *P* value, for each predictor included in the final model of household female *Aedes* mosquito density for the dataset from Posadas, Argentina

Predictor^a^	Level^a^	IRR	IRR 2.5% CI	IRR 97.5% CI	*P*
Number of occupants		1.25	0.98	1.58	0.063
Years spent living in household		0.81	0.55	1.14	0.260
Number of floors		0.76	0.53	1.05	0.110
Number of family cores		1.31	1.08	1.59	0.005*
Distance to nearest household		1.08	0.92	1.27	0.352
Type of lease	Rental	Reference			
	Family	2.68	0.80	9.38	0.110
	Property	0.42	0.20	0.91	0.023*
Frequency of obtaining water	Every other day	Reference			
	Daily	1.45	0.82	2.73	0.212
	Unpredictable	2.96	1.70	5.25	< 0.001*
	Weekly	0.89	0.26	2.90	0.851
Problem obtaining water	No	Reference			
	Yes	0.77	0.47	1.30	0.304
Storing water	No	Reference			
	Yes	1.70	1.02	2.88	0.043*
Using bed nets	No	Reference			
	Yes	0.73	0.41	1.30	0.283
Using window screens	No	Reference			
	Yes	1.79	0.77	3.81	0.146
Other means of protection against mosquitoes	No	Reference			
	Yes	0.52	0.27	0.93	0.031*
Points of entry for mosquitoes into household	No	Reference			
	Yes	2.70	1.05	6.67	0.026*
Presence of vegetation inside household	No	Reference			
	Yes	1.41	0.75	2.90	0.314
Presence of breeding sites	No	Reference			
	Yes	1.55	0.79	3.02	0.192

Estimation of model coefficients was conducted on a total of 92 households (25% of the full dataset). Average proportion of variance explained by random-effects factors, i.e. month and neighborhood, was 0.16 and 0.00, respectively

^a^Predictors that are underlined have an IRR that is significantly different from 1

*Significant at a threshold α = 0.05

## Discussion

Our study of arbovirus vector populations in three study sites located in three Latin American countries, chosen to reflect different eco-epidemiological settings, identified substantial variations in household-level *Aedes* mosquito density, both within and among study sites, and over time. Importantly, we identified several relevant predictors of vector density across study sites, while other factors were more important in certain local contexts. Overall, our findings point to the importance of controlling breeding sites, improving quality of household structure and targeting mosquito surveillance and control to poorer households. 

Our study results indicate that meteorological, climatic and socio-economic variation have all likely contributed to shaping conditions conducive to *Aedes* density in our study across the three sites. Additionally, we found considerable socio-economic differences across the three study sites when we applied a common measure of asset-based household wealth. This is reflected by the sixfold increase in* R*^2^ when accounting for temporal and spatial random-effects (i.e. conditional* R*^2^ compared to marginal* R*^2^) parameters in our multiple regression analyses across study sites. In addition to these spatial and temporal effects, other fine-scale processes are likely to have played a role in shaping individual household responses to dealing with *Aedes* mosquitoes. This is reflected by the differences we observed in the predictive power of variables that we investigated among and across the three study sites.

Analyses of households of Ibagué, Colombia, suggested a major role of variables associated with household occupant characteristics, such as crowding, wealth index and arbovirus knowledge, but also indoor and outdoor household components. Number of occupants in a household, number of floors and distance between households all affected the observed densities of *Aedes* mosquitoes, with a higher degree of crowding associated with higher *Aedes* density. Indeed, an increase in unplanned urbanization, which is typically associated with a rapid increase in human population density, is associated with the creation of new suitable habitats for *Aedes* mosquitoes [17, 25, 42]. We found that wealthier households where occupants displayed higher knowledge of arboviruses were associated with lower *Aedes* density. Household wealth is widely known to affect mosquito vector density, potentially through better access to mosquito control methods [22, 26, 34, 43], and the effect of knowledge about mosquito vectors and arboviruses on household mosquito vector density and arboviral disease risk has also been the subject of many studies [27, 44, 45]. We also found major effects of presence of decorative vegetation and landscape elements around the household. Presence of decorative vegetation and green areas in and around the household and absence of water bodies near the household all led to higher *Aedes* densities. These effects can be explained by the ecology of *Aedes* mosquitoes, which frequently use discarded containers filled with exposed and shady standing water as breeding sites, and use water bodies less frequently, especially those with flowing water and/or situated more than 25 m away from the household [46]. In a study in the USA, managed container habitats in higher wealth neighborhoods, such as those used for decorative plants, and vegetation around households with high abandonment were both associated with higher *Aedes* density [28].

In contrast, analyses of households in Manta, Ecuador, suggested a major role of variables associated with water and waste management, and humidity. Presence of a large water container in or around the household was significantly associated with lower *Aedes* density. On the other hand, humidity, difficulty in obtaining water and weekly waste collection frequency were all significantly associated with larger *Aedes* density. Efficient water management, such as through the use of a dedicated water storage container [47–49], and frequent waste management, which prevents formation of mosquito breeding sites [24, 50], are typically associated with better mosquito control.

Analyses of households in Posadas, Argentina, and across the three sites suggested a major role of a mixture of these effects. In Posadas, households that were owned and those that did not display structural points of entry for mosquitoes displayed lower *Aedes* density, while unpredictable access to water by occupants and storing water were associated to higher *Aedes* density. In analyses across the three study sites, fewer household occupants, higher knowledge of arboviruses by occupants, living fewer years in the household, wealthier households and absence of structural points of entry for mosquitoes into the household were all associated with lower *Aedes* density. Also, across study sites, reporting that water containers were regularly emptied by occupants and absence of decorative vegetation led to lower *Aedes* density. Interestingly, using pesticides in Manta, Ecuador, and using bed nets across study sites showed a positive association with *Aedes* density. These results may either reflect that use of such mosquito control methods is driven by need, for example when mosquitoes are present in high numbers, or that their effect on diurnal mosquitoes is limited. Altogether, our results point to modifiable risk factors related to water and waste management that should be the target of future interventions. These interventions should be prioritized in lower wealth households, regardless of the neighborhood’s overall SES, and include an educational component to raise awareness of risks associated with *Aedes* mosquito presence and potential control methods.

It was not possible to include measures of neighborhood socio-economic profile into our analyses that are both precise and consistent across study sites. For example, we expect that in wealthier neighborhoods, lower household wealth, lower arbovirus knowledge, poorer household structure and weaker water management, which may all increase household *Aedes* density according to results of this study herein and other studies [24, 26, 27, 43, 47, 48], are potentially accompanied with inadequate mosquito control by municipal authorities, which is usually targeted to neighborhoods with higher disease incidence [51]. Wealthier households may also contribute to *Aedes* breeding, potentially through landscape and vegetation elements in and around the household, such as managed water containers for decorative plants and green areas around the household [28]. These effects should be investigated further to discriminate among the neighborhood-level and household-level determinants of *Aedes* breeding and identify appropriate control methods.

Several logistical challenges arose during our study, which led to a delay in the start of the study timeline for two of our study sites. As a result, the study timeline differed across the three sites and the entire timeline for the study lasted 21 months, instead of 12 months. However, we achieved some temporal overlap in study timeline among the three sites for comparability. In addition, two study sites did not have the expected total number of households per neighborhood for high socio-economic areas. For this reason, we sampled households in more than two neighborhoods of high SES in these study sites. This could have led to a difference in spatial resolution between neighborhoods of high versus low SES. However, all neighborhoods were located within close proximity to each other. Our regression analyses incorporated random effects for the month of sampling, nested in sampled neighborhood, nested in study site; therefore, the results of these analyses are unlikely to have been affected by potential temporal and spatial biases.

## Conclusions

Our study identified key variables affecting *Aedes* mosquito density across three study sites reflecting different eco-epidemiological settings in the Latin American region. Our results could help to elucidate some of the complexity in the relationship between meteorological, climatic and socio-economic factors and specific household characteristics that contribute to *Aedes* mosquito vector density, and hence arbovirus risk, in the Latin American region.

## Supplementary Information

**Additional file 1: Household questionnaire survey**. List of all survey questions given to the respondent of each household included in the study, in Spanish.

**Additional file 2: Table S1**. Description of all numerical variables investigated in the study. Mean and standard deviation (SD) of the distribution, number of missing households, mixed-effects regression incidence rate ratio (IRR) values, the* Z* value and the* P* value, for each numerical predictor in simple regression models of household female* Aedes* mosquito density for the entire dataset across study sites.

**Additional file 3: Table S2**. Description of all categorical variables investigated in the study. Number of households at each level, including number of missing households (Ø), mixed-effects regression incidence rate ratio (IRR) values, the* Z* value and the* P* value, for each factorial predictor in simple regression models of household female* Aede*s mosquito density for the entire dataset across study sites.

## Data Availability

All data generated or analysed during this study are included in this published article and its supplementary information files. Raw data files and R scripts are available upon request to the corresponding author.
