# Supplementary material for: Determinants of Aedes mosquito density as an indicator of arbovirus transmission risk in three sites affected by co-circulation of globally spreading arboviruses in Colombia, Ecuador and Argentina
Source: Parasit Vectors. 2021 Sep 19;14:482. doi: 10.1186/s13071-021-04984-z (PMC8451087; doi:10.1186/s13071-021-04984-z)
Supplement: Supplementary file 1 — Additional file 1: Household questionnaire survey. List of all survey questions given to the respondent of each household included in the study, in Spanish. [file 13071_2021_4984_MOESM1_ESM.pdf]

Assessing ZIKV transmission dynamics and mitigation strategies. A multidisciplinary approach.	FORMATO 01: RECOLECCIÓN DE DATOS SOCIO-ECONÓMICOS EN VIVIENDAS		Código:	001
	Macro-Proceso: Centro de Investigación de Vectores Artrópodos	Proceso Interno: Dinámica de Zika	Edición:	03 DV
			Fecha aprobación:	08/03/2018

Fecha de colecta	Código Geográfico	Localidad	Fuente Georeferenciación	Altitud	Colectores	Página
20__ ___ ___<br>AÑO MES DÍA	___ ___ ___ ___<br>PRV DST CRC SBC					1/4

Número de casa	Número de muestras colectadas																

CARACTERÍSTICAS DEL HOGAR

Código	Pregunta	Opciones								
HC1	Años viviendo en esta residencia	años								
HC2	Su casa cuenta con:<br>(Escriba 0/1 para cada item)	a) Radio<br>b) Televisión<br>c) Refrigerador<br>d) Aire acondicionado<br>e) Computador<br>f) Generador, batería, panel solar<br>g) Bicicleta<br>h) Motocicleta<br>i) Auto o Camión	a) ☐<br>b) ☐<br>c) ☐<br>d) ☐<br>e) ☐<br>f) ☐<br>g) ☐<br>h) ☐<br>i) ☐	a) ☐<br>b) ☐<br>c) ☐<br>d) ☐<br>e) ☐<br>f) ☐<br>g) ☐<br>h) ☐<br>i) ☐	a) ☐<br>b) ☐<br>c) ☐<br>d) ☐<br>e) ☐<br>f) ☐<br>g) ☐<br>h) ☐<br>i) ☐	a) ☐<br>b) ☐<br>c) ☐<br>d) ☐<br>e) ☐<br>f) ☐<br>g) ☐<br>h) ☐<br>i) ☐	a) ☐<br>b) ☐<br>c) ☐<br>d) ☐<br>e) ☐<br>f) ☐<br>g) ☐<br>h) ☐<br>i) ☐	a) ☐<br>b) ☐<br>c) ☐<br>d) ☐<br>e) ☐<br>f) ☐<br>g) ☐<br>h) ☐<br>i) ☐	a) ☐<br>b) ☐<br>c) ☐<br>d) ☐<br>e) ☐<br>f) ☐<br>g) ☐<br>h) ☐<br>i) ☐	a) ☐<br>b) ☐<br>c) ☐<br>d) ☐<br>e) ☐<br>f) ☐<br>g) ☐<br>h) ☐<br>i) ☐
HC3	Su vivienda es	a) Propia<br>b) Arrendada<br>c) Familiar	a) ☐<br>b) ☐<br>c) ☐	a) ☐<br>b) ☐<br>c) ☐	a) ☐<br>b) ☐<br>c) ☐	a) ☐<br>b) ☐<br>c) ☐	a) ☐<br>b) ☐<br>c) ☐	a) ☐<br>b) ☐<br>c) ☐	a) ☐<br>b) ☐<br>c) ☐	a) ☐<br>b) ☐<br>c) ☐

CARACTERÍSTICAS OBSERVADAS EN EL HOGAR

Código	Pregunta	Opciones								
OB1	Coordenadas GPS	Latitud Y (Norte-Sur)								
		Longitud X (Oeste)								
OB2	Tipo de residencia	a) Casa independiente<br>b) Edificio	a) ☐<br>b) ☐	a) ☐<br>b) ☐	a) ☐<br>b) ☐	a) ☐<br>b) ☐	a) ☐<br>b) ☐	a) ☐<br>b) ☐	a) ☐<br>b) ☐	a) ☐<br>b) ☐
OB3	¿Cuántos pisos y núcleos familiares tiene el hogar?	Pisos								
		Núcleos familiares								
OB4	Distancia en metros de la casa más cercana	Metros								
OB5	¿Tiene electricidad?	a) No<br>b) Si	a) ☐<br>b) ☐	a) ☐<br>b) ☐	a) ☐<br>b) ☐	a) ☐<br>b) ☐	a) ☐<br>b) ☐	a) ☐<br>b) ☐	a) ☐<br>b) ☐	a) ☐<br>b) ☐
OB6	Tipo de material del techo de la casa	a) Temporal<br>bambú, paja, hierba, heno, hojas<br>b) Permanente<br>Madera, concreto, ladrillo, piedra, hierro/ aluminio galvanizado, fibrocemento, otras hojas de metal	a) ☐<br>b) ☐	a) ☐<br>b) ☐	a) ☐<br>b) ☐	a) ☐<br>b) ☐	a) ☐<br>b) ☐	a) ☐<br>b) ☐	a) ☐<br>b) ☐	a) ☐<br>b) ☐
OB7	Material principal del piso de la casa	a) Natural: tierra, arena, arcilla<br>b) Rudimentario: madera, palma, bambú<br>c) Terminado: Parqué o madera lacada, vinil, asfalto, cerámica, cemento, baldosa.	a) ☐<br>b) ☐<br>c) ☐	a) ☐<br>b) ☐<br>c) ☐	a) ☐<br>b) ☐<br>c) ☐	a) ☐<br>b) ☐<br>c) ☐	a) ☐<br>b) ☐<br>c) ☐	a) ☐<br>b) ☐<br>c) ☐	a) ☐<br>b) ☐<br>c) ☐	a) ☐<br>b) ☐<br>c) ☐
OB8	Material principal de las paredes de la casa	a) Temporal<br>bambú, paja, hierba, caña, tierra, materiales rescatados, heno, hojas<br>b) Permanente<br>Madera, concreto, ladrillo, piedra, hierro/ aluminio galvanizado, fibrocemento, otras hojas de metal	a) ☐<br>b) ☐	a) ☐<br>b) ☐	a) ☐<br>b) ☐	a) ☐<br>b) ☐	a) ☐<br>b) ☐	a) ☐<br>b) ☐	a) ☐<br>b) ☐	a) ☐<br>b) ☐
OB9	Presencia de contenedores que puedan acumular agua en el jardín	a) No<br>b) Si	a) ☐<br>b) ☐	a) ☐<br>b) ☐	a) ☐<br>b) ☐	a) ☐<br>b) ☐	a) ☐<br>b) ☐	a) ☐<br>b) ☐	a) ☐<br>b) ☐	a) ☐<br>b) ☐
OB10	Tipo de contenedores	a) Tanques de agua sin protección<br>b) Contenedores grandes usados para almacenamiento de agua<br>c) Botellas<br>d) Otros contenedores	a) ☐<br>b) ☐<br>c) ☐<br>d) ☐	a) ☐<br>b) ☐<br>c) ☐<br>d) ☐	a) ☐<br>b) ☐<br>c) ☐<br>d) ☐	a) ☐<br>b) ☐<br>c) ☐<br>d) ☐	a) ☐<br>b) ☐<br>c) ☐<br>d) ☐	a) ☐<br>b) ☐<br>c) ☐<br>d) ☐	a) ☐<br>b) ☐<br>c) ☐<br>d) ☐	a) ☐<br>b) ☐<br>c) ☐<br>d) ☐

DISPONIBILIDAD DE AGUA

Código	Pregunta	Opciones								
WS1	Principal fuente de agua potable para tomar	a) Agua entubada<br>b) Agua de pozo<br>c) Agua de manantial<br>d) Lluvia<br>e) Embotellada<br>f) Otra (especificar)	a) ☐<br>b) ☐<br>c) ☐<br>d) ☐<br>e) ☐<br>f) ____	a) ☐<br>b) ☐<br>c) ☐<br>d) ☐<br>e) ☐<br>f) ____	a) ☐<br>b) ☐<br>c) ☐<br>d) ☐<br>e) ☐<br>f) ____	a) ☐<br>b) ☐<br>c) ☐<br>d) ☐<br>e) ☐<br>f) ____	a) ☐<br>b) ☐<br>c) ☐<br>d) ☐<br>e) ☐<br>f) ____	a) ☐<br>b) ☐<br>c) ☐<br>d) ☐<br>e) ☐<br>f) ____	a) ☐<br>b) ☐<br>c) ☐<br>d) ☐<br>e) ☐<br>f) ____	a) ☐<br>b) ☐<br>c) ☐<br>d) ☐<br>e) ☐<br>f) ____
WS2	Principal fuente de agua usada para otros propósitos (limpieza, lavado y cocina)	a) Agua entubada<br>b) Agua de pozo<br>c) Agua de manantial<br>d) Lluvia<br>e) Embotellada<br>f) Otra (especificar)	a) ☐<br>b) ☐<br>c) ☐<br>d) ☐<br>e) ☐<br>f) ____	a) ☐<br>b) ☐<br>c) ☐<br>d) ☐<br>e) ☐<br>f) ____	a) ☐<br>b) ☐<br>c) ☐<br>d) ☐<br>e) ☐<br>f) ____	a) ☐<br>b) ☐<br>c) ☐<br>d) ☐<br>e) ☐<br>f) ____	a) ☐<br>b) ☐<br>c) ☐<br>d) ☐<br>e) ☐<br>f) ____	a) ☐<br>b) ☐<br>c) ☐<br>d) ☐<br>e) ☐<br>f) ____	a) ☐<br>b) ☐<br>c) ☐<br>d) ☐<br>e) ☐<br>f) ____	a) ☐<br>b) ☐<br>c) ☐<br>d) ☐<br>e) ☐<br>f) ____

CONICET INSTITUTO DE BIOLOGÍA SUBTROPICAL · UNaM Universidad Nacional de Misiones · Universidad de los Andes Colombia · Uleam Universidad Laica Eloy Alfaro de Manabí · Instituto Nacional de Investigación en Salud Pública Dr. Leopoldo Izquieta Pérez · YORK University · University Health Network · uOttawa · IDRC CRDI International Development Research Centre / Centre de recherches pour le développement international · CIHR IRSC Canadian Institutes of Health Research / Instituts de recherche en santé du Canada

Esta prohibida la reproducción total o parcial de este documento. La información contenida es de propiedad del Instituto Nacional de Investigación en Salud Pública Dr. Leopoldo Izquieta Pérez

Fecha de colecta	Código Geográfico	Localidad	Fuente Georeferenciación	Altitud	Colectores	Página
20__ __ __ AÑO MES DÍA	__ __ __ __ PRV DST CRC SBC					2/4

	Número de casa	Número de muestras colectadas								
WS3	¿Tiene problemas de abastecimiento de agua?	a) No (ir a WS5)<br>b) Si	a)<br>b)	a)<br>b)	a)<br>b)	a)<br>b)	a)<br>b)	a)<br>b)	a)<br>b)	a)<br>b)
WS4	¿Con qué frecuencia tiene el servicio de agua en la casa?	a) Más de una vez al día<br>b) Diario<br>c) Pasando un día<br>d) Semanal<br>e) Impredecible	a)<br>b)<br>c)<br>d)<br>e)	a)<br>b)<br>c)<br>d)<br>e)	a)<br>b)<br>c)<br>d)<br>e)	a)<br>b)<br>c)<br>d)<br>e)	a)<br>b)<br>c)<br>d)<br>e)	a)<br>b)<br>c)<br>d)<br>e)	a)<br>b)<br>c)<br>d)<br>e)	a)<br>b)<br>c)<br>d)<br>e)
WS5	¿Almacena agua?	a) No (fin de la sección)<br>b) Si	a)<br>b)	a)<br>b)	a)<br>b)	a)<br>b)	a)<br>b)	a)<br>b)	a)<br>b)	a)<br>b)
WS6	¿Para qué propósito almacena agua? (seleccione todos los que aplican)	a) Tomar<br>b) Limpieza<br>c) Lavado<br>d) Cocinar<br>e) Otra (especificar)	a)<br>b)<br>c)<br>d)<br>e)____	a)<br>b)<br>c)<br>d)<br>e)____	a)<br>b)<br>c)<br>d)<br>e)____	a)<br>b)<br>c)<br>d)<br>e)____	a)<br>b)<br>c)<br>d)<br>e)____	a)<br>b)<br>c)<br>d)<br>e)____	a)<br>b)<br>c)<br>d)<br>e)____	a)<br>b)<br>c)<br>d)<br>e)____
WS7	¿Cepilla los contenedores después de remover el agua?	a) No<br>b) Si	a)<br>b)	a)<br>b)	a)<br>b)	a)<br>b)	a)<br>b)	a)<br>b)	a)<br>b)	a)<br>b)
**MANEJO DE DESECHOS**										
WD1	¿Cómo se eliminan las aguas negras en su hogar?	a) Alcantarilla<br>b) Letrina<br>c) Pozo séptico<br>d) Otro (especificar)	a)<br>b)<br>c)<br>d)____	a)<br>b)<br>c)<br>d)____	a)<br>b)<br>c)<br>d)____	a)<br>b)<br>c)<br>d)____	a)<br>b)<br>c)<br>d)____	a)<br>b)<br>c)<br>d)____	a)<br>b)<br>c)<br>d)____	a)<br>b)<br>c)<br>d)____
WD2	¿Cómo elimina la basura en su hogar?	a) recolección municipal o privada<br>b) desecho en una ubicación dentro de la propiedad<br>c) desecho en una ubicación fuera de la propiedad<br>d) desecho irregular<br>e) Otro (especificar)	a)<br>b)<br>c)<br>d)<br>e)____	a)<br>b)<br>c)<br>d)<br>e)____	a)<br>b)<br>c)<br>d)<br>e)____	a)<br>b)<br>c)<br>d)<br>e)____	a)<br>b)<br>c)<br>d)<br>e)____	a)<br>b)<br>c)<br>d)<br>e)____	a)<br>b)<br>c)<br>d)<br>e)____	a)<br>b)<br>c)<br>d)<br>e)____
WD3	Frecuencia de recolección de basura	a) Diario<br>b) Pasando un día<br>c) Semanal<br>d) Mensual<br>e) Impredecible	a)<br>b)<br>c)<br>d)<br>e)	a)<br>b)<br>c)<br>d)<br>e)	a)<br>b)<br>c)<br>d)<br>e)	a)<br>b)<br>c)<br>d)<br>e)	a)<br>b)<br>c)<br>d)<br>e)	a)<br>b)<br>c)<br>d)<br>e)	a)<br>b)<br>c)<br>d)<br>e)	a)<br>b)<br>c)<br>d)<br>e)
**PREVENCIÓN DE MOSQUITO Y MEDIDAS DE CONTROL**										
MP1	¿Utiliza insecticida en su hogar por su propia cuenta?	a) No (ir a MP5)<br>b) Si	a)<br>b)	a)<br>b)	a)<br>b)	a)<br>b)	a)<br>b)	a)<br>b)	a)<br>b)	a)<br>b)
MP2	¿Con qué frecuencia?									
MP3	¿Cuándo fue la última vez? (DD/MM/AAAA)		__/__/____	__/__/____	__/__/____	__/__/____	__/__/____	__/__/____	__/__/____	__/__/____
MP4	¿Cuál fue el producto utilizado?		________	________	________	________	________	________	________	________
MP5	¿Cuándo fue la última visita del Ministerio/Secretaría de Salud para fumigación/abatización? (DD/MM/AAAA)	Fumigación	__/__/____	__/__/____	__/__/____	__/__/____	__/__/____	__/__/____	__/__/____	__/__/____
		Abatización	__/__/____	__/__/____	__/__/____	__/__/____	__/__/____	__/__/____	__/__/____	__/__/____
MP6	¿Cuál de las siguientes medidas utiliza para prevención de picadura de mosquito?	a) Repelente<br>b) Repelente en espiral<br>c) Aerosol<br>d) Toldo<br>e) Tratamiento de cortinas con insecticida<br>f) Malla para ventanas<br>g) Cobertura para contenedores de agua<br>h) Lavado y limpieza de contenedores<br>Frecuencia DD/MM/AAA<br>i) Vaciado de contenedores de agua<br>Frecuencia DD/MM/AAA<br>j) Manejo de desechos en la casa<br>k) Mata moscas<br>l) Ropa protectora<br>m) Otro (especificar)	a)<br>b)<br>c)<br>d)<br>e)<br>f)<br>g)<br>h)<br>__/__/____<br>i)<br>__/__/____<br>j)<br>k)<br>l)<br>m)____	a)<br>b)<br>c)<br>d)<br>e)<br>f)<br>g)<br>h)<br>__/__/____<br>i)<br>__/__/____<br>j)<br>k)<br>l)<br>m)____	a)<br>b)<br>c)<br>d)<br>e)<br>f)<br>g)<br>h)<br>__/__/____<br>i)<br>__/__/____<br>j)<br>k)<br>l)<br>m)____	a)<br>b)<br>c)<br>d)<br>e)<br>f)<br>g)<br>h)<br>__/__/____<br>i)<br>__/__/____<br>j)<br>k)<br>l)<br>m)____	a)<br>b)<br>c)<br>d)<br>e)<br>f)<br>g)<br>h)<br>__/__/____<br>i)<br>__/__/____<br>j)<br>k)<br>l)<br>m)____	a)<br>b)<br>c)<br>d)<br>e)<br>f)<br>g)<br>h)<br>__/__/____<br>i)<br>__/__/____<br>j)<br>k)<br>l)<br>m)____	a)<br>b)<br>c)<br>d)<br>e)<br>f)<br>g)<br>h)<br>__/__/____<br>i)<br>__/__/____<br>j)<br>k)<br>l)<br>m)____	a)<br>b)<br>c)<br>d)<br>e)<br>f)<br>g)<br>h)<br>__/__/____<br>i)<br>__/__/____<br>j)<br>k)<br>l)<br>m)____

Esta prohibida la reproducción total o parcial de este documento. La información contenida es de propiedad del Instituto Nacional de Investigación en Salud Pública Dr. Leopoldo Izquieta Pérez

Assessing ZIKV transmission dynamics and mitigation strategies. A multidisciplinary approach.	FORMATO 01: RECOLECCIÓN DE DATOS SOCIO-ECONÓMICOS EN VIVIENDAS		Código:	001
	Macro-Proceso: Centro de Investigación de Vectores Artrópodos	Proceso Interno: Dinámica de Zika	Edición:	03 DV
			Fecha aprobación:	08/03/2018

Fecha de colecta	Código Geográfico	Localidad	Fuente Georeferenciación	Altitud	Colectores	Página
20__ __ AÑO __ __ MES __ __ DÍA	__ __ PRV __ __ DST __ __ CRC __ __ SBC					3/4

Número de casa	Número de muestras colectadas									
**MP7**	**¿Utiliza malla protectora en ventanas y puertas?**	a) No<br>b) Si	a) b)	a) b)	a) b)	a) b)	a) b)	a) b)	a) b)	a) b)
**MP8**	**¿En dónde está ubicado el baño?**	a) Dentro del hogar<br>b) Fuera del hogar<br>c) Baño comunitario<br>d) Otro (especificar)	a) b) c) d)___	a) b) c) d)___	a) b) c) d)___	a) b) c) d)___	a) b) c) d)___	a) b) c) d)___	a) b) c) d)___	a) b) c) d)___
**MP9**	**Existen puntos de ingreso de mosquitos aparte de ventanas y puertas**	a) No<br>b) Si	a) b)	a) b)	a) b)	a) b)	a) b)	a) b)	a) b)	a) b)
**MP10**	**¿Tiene área verde alrededor de la propiedad?**	a) No<br>b) Si	a) b)	a) b)	a) b)	a) b)	a) b)	a) b)	a) b)	a) b)
**MP11**	**¿Existen fuentes de agua cercanas al hogar? Especifique la distancia en metros**	a) Ninguna<br>b) Estanque<br>c) Lago<br>d) Zanja<br>e) Canal<br>f) Río	a) b) c) d) e) f)	a) b) c) d) e) f)	a) b) c) d) e) f)	a) b) c) d) e) f)	a) b) c) d) e) f)	a) b) c) d) e) f)	a) b) c) d) e) f)	a) b) c) d) e) f)
**MP12**	**¿Tiene vegetación (natural o cultivada) en su propiedad?**	a) No<br>b) Si	a) b)	a) b)	a) b)	a) b)	a) b)	a) b)	a) b)	a) b)
**MP13**	**¿Tiene plantas en macetas en partes altas?**	a) No<br>b) Si	a) b)	a) b)	a) b)	a) b)	a) b)	a) b)	a) b)	a) b)
**MP14**	**¿Qué tipo de vegetación tiene?**	a) Hierba<br>b) Arbustos<br>c) Viñedos<br>d) Huertos<br>e) Árboles<br>f) Otro (especificar)	a) b) c) d) e) f)___	a) b) c) d) e) f)___	a) b) c) d) e) f)___	a) b) c) d) e) f)___	a) b) c) d) e) f)___	a) b) c) d) e) f)___	a) b) c) d) e) f)___	a) b) c) d) e) f)___
**MP15**	**¿Existen criaderos con larvas en la propiedad?**	a) No<br>b) Si	a) b)	a) b)	a) b)	a) b)	a) b)	a) b)	a) b)	a) b)
**DEMOGRAFÍA DEL HOGAR**										
**HD1**	**¿Cuántas personas viven normalmente en el hogar?**	a) Adultos<br>b) Niños	a) b)	a) b)	a) b)	a) b)	a) b)	a) b)	a) b)	a) b)
**HD2**	**¿Cuántas personas trabajan/ estudian en el hogar?**	Trabajan<br>Estudian								
**HD3**	**¿Cuántas personas saben leer o escribir?**									
**HD4**	**¿Cuántas personas son mayores de edad?**									
**RESPUESTAS DE CONOCIMIENTO (DENGUE)**										
**RK1**	**¿Ha escuchado del dengue?**	a) No (ir a RK6)<br>b) Si	a) b)	a) b)	a) b)	a) b)	a) b)	a) b)	a) b)	a) b)
**RK2**	**¿Algún miembro de la familia ha tenido alguna vez dengue?**	a) No (ir a RK4)<br>b) Si<br>¿Cuándo? (mm/aaaa)	a) b) __/____	a) b) __/____	a) b) __/____	a) b) __/____	a) b) __/____	a) b) __/____	a) b) __/____	a) b) __/____
**RK3**	**¿Cuántas personas en el hogar han tenido dengue en el último año?**									
**RK4**	**¿Cuántas personas en el hogar han solicitado atención médica por infección con dengue en el último año?**									
**RESPUESTAS DE CONOCIMIENTO (CHIKUNGUNYA)**										
**RK5**	**¿Ha escuchado de chikungunya?**	a) No (ir a RK11)<br>b) Si	a) b)	a) b)	a) b)	a) b)	a) b)	a) b)	a) b)	a) b)
**RK6**	**¿Algún miembro de la familia ha tenido alguna vez chikungunya?**	a) No (ir a RK9)<br>b) Si<br>¿Cuándo? (mm/aaaa)	a) b) __/____	a) b) __/____	a) b) __/____	a) b) __/____	a) b) __/____	a) b) __/____	a) b) __/____	a) b) __/____
**RK7**	**¿Cuántas personas en el hogar han tenido chikungunya en el último año?**									

Esta prohibida la reproducción total o parcial de este documento. La información contenida es de propiedad del Instituto Nacional de Investigación en Salud Pública Dr. Leopoldo Izquieta Pérez

Assessing ZIKV transmission dynamics and mitigation strategies. A multidisciplinary approach.	FORMATO 01: RECOLECCIÓN DE DATOS SOCIO-ECONÓMICOS EN VIVIENDAS		Código:	001
	Macro-Proceso: Centro de Investigación de Vectores Artrópodos	Proceso Interno: Dinámica de Zika	Edición:	03 DV
			Fecha aprobación:	08/03/2018

Fecha de colecta	Código Geográfico	Localidad	Fuente Georeferenciación	Altitud	Colectores	Página
20__ __ __ (AÑO MES DÍA)	__ __ __ __ (PRV DST CRC SBC)					4/4

		Número de casa	Número de muestras colectadas							
**RK08**	**¿Cuántas personas en el hogar han solicitado atención médica por infección con chikungunya en el último año?**									

RESPUESTAS DE CONOCIMIENTO (ZIKA)

Código	Pregunta	Opciones	1	2	3	4	5	6	7	8
**RK09**	**¿Ha escuchado del zika?**	a) No<br>b) Si	a) ☐<br>b) ☐	a) ☐<br>b) ☐	a) ☐<br>b) ☐	a) ☐<br>b) ☐	a) ☐<br>b) ☐	a) ☐<br>b) ☐	a) ☐<br>b) ☐	a) ☐<br>b) ☐
**RK10**	**¿Ha tenido alguna vez zika?**	a) No (ir a RK14)<br>b) Si<br>¿Cuándo? (mm / aaaa)	a) ☐<br>b) ☐<br>__/____	a) ☐<br>b) ☐<br>__/____	a) ☐<br>b) ☐<br>__/____	a) ☐<br>b) ☐<br>__/____	a) ☐<br>b) ☐<br>__/____	a) ☐<br>b) ☐<br>__/____	a) ☐<br>b) ☐<br>__/____	a) ☐<br>b) ☐<br>__/____
**RK11**	**¿Cuántas personas en el hogar han tenido zika en el último año?**									
**RK12**	**¿Cuántas personas en el hogar han solicitado atención médica por infección con zika en el último año?**									
**RK13**	**Como se transmite el dengue / zika / chikungunya**	a) Identifica los mosquitos como modo principal de transmisión<br>b) No identifica los mosquitos como un modo de transmisión, o lo identifica como un modo secundario de transmisión (Especificar)	a) ☐<br>b) ☐<br>________	a) ☐<br>b) ☐<br>________	a) ☐<br>b) ☐<br>________	a) ☐<br>b) ☐<br>________	a) ☐<br>b) ☐<br>________	a) ☐<br>b) ☐<br>________	a) ☐<br>b) ☐<br>________	a) ☐<br>b) ☐<br>________
**RK14**	**¿Cómo se protege de la infección con dengue / zika / chikungunya? (Seleccione toda las que corresponden)**	a) Nada<br>b) Elimina agua estancada sin protección dentro/cerca de la casa<br>c) Se protege contra los mosquitos<br>d) Otro (especificar)	a) ☐<br>b) ☐<br>c) ☐<br>d) ☐	a) ☐<br>b) ☐<br>c) ☐<br>d) ☐	a) ☐<br>b) ☐<br>c) ☐<br>d) ☐	a) ☐<br>b) ☐<br>c) ☐<br>d) ☐	a) ☐<br>b) ☐<br>c) ☐<br>d) ☐	a) ☐<br>b) ☐<br>c) ☐<br>d) ☐	a) ☐<br>b) ☐<br>c) ☐<br>d) ☐	a) ☐<br>b) ☐<br>c) ☐<br>d) ☐
**RK15**	**¿Lleva a cabo la comunidad (barrio/conjunto) actividades para prevenir y controlar dengue / chikungunya / zika?**	a) No<br>b) Si	a) ☐<br>b) ☐	a) ☐<br>b) ☐	a) ☐<br>b) ☐	a) ☐<br>b) ☐	a) ☐<br>b) ☐	a) ☐<br>b) ☐	a) ☐<br>b) ☐	a) ☐<br>b) ☐
**RK16**	**¿Su familia ha participado en actividades para prevenir y controlar dengue / chikungunya / zika?**	a) No<br>b) Si	a) ☐<br>b) ☐	a) ☐<br>b) ☐	a) ☐<br>b) ☐	a) ☐<br>b) ☐	a) ☐<br>b) ☐	a) ☐<br>b) ☐	a) ☐<br>b) ☐	a) ☐<br>b) ☐
**RK17**	**¿Cuáles son los principales problemas en su barrio que contribuyen a la transmisión de dengue / chikungunya / Zika? (Seleccione todos los que aplican)**	a) Presencia de contenedores que almacenan agua<br>b) Alta densidad de población humana<br>c) Grandes poblaciones de mosquitos<br>d) Otro (especificar)	a) ☐<br>b) ☐<br>c) ☐<br>d) ____	a) ☐<br>b) ☐<br>c) ☐<br>d) ____	a) ☐<br>b) ☐<br>c) ☐<br>d) ____	a) ☐<br>b) ☐<br>c) ☐<br>d) ____	a) ☐<br>b) ☐<br>c) ☐<br>d) ____	a) ☐<br>b) ☐<br>c) ☐<br>d) ____	a) ☐<br>b) ☐<br>c) ☐<br>d) ____	a) ☐<br>b) ☐<br>c) ☐<br>d) ____

CONICET INSTITUTO DE BIOLOGÍA SUBTROPICAL · UNIVERSIDAD NACIONAL DE MISIONES · Universidad de los Andes Colombia · Uleam UNIVERSIDAD LAICA ELOY ALFARO DE MANABÍ · INSTITUTO NACIONAL DE INVESTIGACIÓN EN SALUD PÚBLICA Dr. Leopoldo Izquieta Pérez · YORK UNIVERSITÉ UNIVERSITY · University Health Network · uOttawa · IDRC CRDI International Development Research Centre / Centre de recherches pour le développement international · CIHR IRSC Canadian Institutes of Health Research / Instituts de recherche en santé du Canada

Esta prohibida la reproducción total o parcial de este documento. La información contenida es de propiedad del Instituto Nacional de Investigación en Salud Pública Dr. Leopoldo Izquieta Pérez
