# Supplementary material for: Determinants of Aedes mosquito density as an indicator of arbovirus transmission risk in three sites affected by co-circulation of globally spreading arboviruses in Colombia, Ecuador and Argentina
Source: Parasit Vectors. 2021 Sep 19;14:482. doi: 10.1186/s13071-021-04984-z (PMC8451087; doi:10.1186/s13071-021-04984-z)
Supplement: Supplementary file 2 — Additional file 2: Table S1. Description of all numerical variables investigated in the study. Mean and standard deviation (SD) of the distribution, number of missing households, mixed-effects regression incidence rate ratio (IRR) values, the Z value and the P value, for each numerical predictor in simple regression models of household female Aedes mosquito density for the entire dataset across study sites. [file 13071_2021_4984_MOESM2_ESM.docx]

| **Variable** | **Mean [SD]** | **Number of households missing (%), N = 1086** | **IRR** | **z** | ***P*** |
| --- | --- | --- | --- | --- | --- |
| Proportion lowest wealth households (%) | 33.33333 [21.51557] | 0 (0.00) | 0.99 | -2.01 | 0.045 |
| Altitude (m) | 866.465116 [406.0538905] | 613 (56.45) | 0.97 | -0.17 | 0.861 |
| Humidity (%) | 69.18126 [12.5617577] | 236 (21.73) | 1.07 | 1.40 | 0.163 |
| Temperature (°C) | 24.985229 [7.3110037] | 240 (22.10) | 0.96 | -0.87 | 0.385 |
| Number of occupants | 4.220441 [2.3499083] | 88 (8.10) | 1.08 | 3.66 | < 0.001 |
| Arbovirus knowledge (6 questions) | 4.75985 [1.0044603] | 20 (1.84) | 0.90 | -4.06 | < 0.001 |
| Number of years spent in household | 18.494405 [21.9384373] | 103 (9.48) | 1.05 | 2.76 | 0.006 |
| Number of floors | 1.525725 [0.9955625] | 17 (1.57) | 1.01 | 0.19 | 0.849 |
| Number of family cores | 1.395178 [1.1670619] | 132 (12.15) | 0.99 | -0.35 | 0.726 |
| Distance from next household (m) | 6.77861 [9.0590008] | 151 (13.90) | 1.06 | 1.98 | 0.048 |
| Number of occupants who work | 1.910377 [1.5055722] | 26 (2.39) | 1.01 | 0.64 | 0.519 |
| Number of occupants who study | 1.262154 [1.437936] | 37 (3.41) | 1.07 | 2.84 | 0.005 |
| Number of occupants who can read and write | 3.396975 [2.0700299] | 28 (2.58) | 1.09 | 4.24 | < 0.001 |
