# Supplementary material for: Determinants of Aedes mosquito density as an indicator of arbovirus transmission risk in three sites affected by co-circulation of globally spreading arboviruses in Colombia, Ecuador and Argentina
Source: Parasit Vectors. 2021 Sep 19;14:482. doi: 10.1186/s13071-021-04984-z (PMC8451087; doi:10.1186/s13071-021-04984-z)
Supplement: Supplementary file 3 — Additional file 3: Table S2. Description of all categorical variables investigated in the study. Number of households at each level, including number of missing households (Ø), mixed-effects regression incidence rate ratio (IRR) values, the Z value and the P value, for each factorial predictor in simple regression models of household female Aedes mosquito density for the entire dataset across study sites. [file 13071_2021_4984_MOESM3_ESM.docx]

| **Factor** | **Factor level** | **Number of households (%), N = 1086** | **IRR** | **z** | ***P*** |
| --- | --- | --- | --- | --- | --- |
| Perceived neighborhood socio-economic status | High | 526 (48.43) | 1.06 | 0.27 | 0.790 |
|  | Low | 560 (51.57) | 1.11 | 0.38 | 0.701 |
| Household wealth index | Lowest | 362 (33.33) | 1.16 | 0.74 | 0.457 |
|  | Middle | 362 (33.33) | 1.04 | 0.61 | 0.545 |
|  | Highest | 362 (33.33) | 0.84 | -2.30 | 0.022 |
| Type of lease agreement | Rental | 135 (12.43) | 1.19 | 0.85 | 0.397 |
|  | Family owned | 88 (8.1) | 1.03 | 0.24 | 0.810 |
|  | Property | 828 (76.24) | 1.19 | 0.85 | 0.397 |
|  | Ø | 35 (3.22) | Ø | Ø | Ø |
| Type of floor materials | Temporary | 8 (0.74) | 2.41 | 2.65 | 0.008 |
|  | Permanent | 1076 (99.08) | 0.46 | -2.78 | 0.005 |
|  | Ø | 2 (0.18) | Ø | Ø | Ø |
| Type of roof materials | Temporary | 32 (2.95) | 1.63 | 2.04 | 0.041 |
|  | Permanent | 1050 (96.69) | 0.68 | -2.44 | 0.015 |
|  | Ø | 4 (0.37) | Ø | Ø | Ø |
| Type of wall materials | Temporary | 19 (1.75) | 0.89 | -0.38 | 0.704 |
|  | Permanent | 1064 (97.97) | 1.26 | 0.89 | 0.376 |
|  | Ø | 3 (0.28) | Ø | Ø | Ø |
| Presence of container in household yard | No | 646 (59.48) | 1.11 | 0.60 | 0.550 |
|  | Yes | 433 (39.87) | 1.01 | 0.21 | 0.833 |
|  | Ø | 7 (0.64) | Ø | Ø | Ø Ø |
| Presence of tank | No | 583 (53.68) | 1.11 | 0.56 | 0.579 |
|  | Yes | 334 (30.76) | 1.02 | 0.37 | 0.709 |
|  | Ø | 169 (15.56) | Ø | Ø | Ø |
| Presence of large water container | No | 503 (46.32) | 1.21 | 0.98 | 0.327 |
|  | Yes | 416 (38.31) | 0.83 | -2.83 | 0.005 |
|  | Ø | 167 (15.38) | Ø | Ø | Ø |
| Presence of bottle | No | 891 (82.04) | 1.13 | 0.69 | 0.493 |
|  | Yes | 25 (2.3) | 0.76 | -1.60 | 0.109 |
|  | Ø | 170 (15.65) | Ø | Ø | Ø |
| Main source of drinking water | Running water | 759 (69.89) | 1.12 | 0.63 | 0.531 |
|  | Springwater | 9 (0.83) | 0.17 | -1.53 | 0.125 |
|  | Well water | 12 (1.1) | 0.53 | -1.51 | 0.131 |
|  | Bottled water | 285 (26.24) | 1.06 | 0.72 | 0.469 |
|  | Rainwater | 2 (0.18) | 1.83 | 0.97 | 0.332 |
|  | Other type | 18 (1.66) | 1.12 | 0.63 | 0.531 |
|  | Ø | 1 (0.09) | Ø | Ø | Ø |
| Main source of water for other uses | Running water | 1037 (95.49) | 1027565.00 | 0.30 | 0.767 |
|  | Springwater | 1 (0.09) | 0.00 | -0.29 | 0.769 |
|  | Well water | 16 (1.47) | 1044619.00 | 0.30 | 0.767 |
|  | Bottled water | 12 (1.1) | 235633.50 | 0.26 | 0.791 |
|  | Rainwater | 6 (0.55) | 1049186.00 | 0.30 | 0.767 |
|  | Ø | 14 (1.29) | Ø | Ø | Ø |
| Problems obtaining water | No | 793 (73.02) | 0.99 | -0.04 | 0.968 |
|  | Yes | 288 (26.52) | 1.50 | 6.05 | < 0.001 |
|  | Ø | 5 (0.46) | Ø | Ø | Ø |
| Frequency of obtaining water | Daily | 494 (45.49) | 1.00 | 0.02 | 0.983 |
|  | Every other day | 98 (9.02) | 1.30 | 1.46 | 0.145 |
|  | Weekly | 31 (2.85) | 1.30 | 1.46 | 0.145 |
|  | Unpredictable | 48 (4.42) | 1.34 | 1.68 | 0.094 |
|  | Ø | 415 (38.21) | Ø | Ø | Ø |
| Storing water | No | 659 (60.68) | 1.08 | 0.46 | 0.644 |
|  | Yes | 425 (39.13) | 1.08 | 1.05 | 0.295 |
|  | Ø | 2 (0.18) | Ø | Ø | Ø |
| Storing water to drink | No | 744 (68.51) | 1.13 | 0.64 | 0.524 |
|  | Yes | 157 (14.46) | 1.13 | 1.52 | 0.128 |
|  | Ø | 185 (17.03) | Ø | Ø | Ø |
| Storing water to clean | No | 367 (33.79) | 1.18 | 0.85 | 0.397 |
|  | Yes | 534 (49.17) | 0.97 | -0.49 | 0.621 |
|  | Ø | 185 (17.03) | Ø | Ø | Ø |
| Storing water to wash | No | 425 (39.13) | 1.16 | 0.76 | 0.449 |
|  | Yes | 476 (43.83) | 1.00 | -0.03 | 0.973 |
|  | Ø | 185 (17.03) | Ø | Ø | Ø |
| Storing water to cook | No | 655 (60.31) | 1.12 | 0.56 | 0.576 |
|  | Yes | 246 (22.65) | 1.14 | 1.95 | 0.052 |
|  | Ø | 185 (17.03) | Ø | Ø | Ø |
| Brushing containers after removing water | No | 125 (11.51) | 0.93 | -0.30 | 0.761 |
|  | Yes | 632 (58.2) | 1.43 | 3.29 | 0.001 |
|  | Ø | 329 (30.29) | Ø | Ø | Ø |
| Using insecticide | No | 518 (47.7) | 1.15 | 0.76 | 0.445 |
|  | Yes | 558 (51.38) | 0.95 | -0.94 | 0.348 |
|  | Ø | 10 (0.92) | Ø | Ø | Ø |
| Using mosquito repellent | No | 594 (54.7) | 1.18 | 0.93 | 0.354 |
|  | Yes | 490 (45.12) | 0.88 | -2.45 | 0.014 |
|  | Ø | 2 (0.18) | Ø | Ø | Ø |
| Using mosquito coil | No | 752 (69.24) | 1.09 | 0.48 | 0.634 |
|  | Yes | 333 (30.66) | 1.07 | 1.02 | 0.309 |
|  | Ø | 1 (0.09) | Ø | Ø | Ø |
| Using aerosol | No | 828 (76.24) | 1.11 | 0.57 | 0.569 |
|  | Yes | 257 (23.66) | 1.03 | 0.46 | 0.645 |
|  | Ø | 1 (0.09) | Ø | Ø | Ø |
| Using bed net | No | 867 (79.83) | 1.05 | 0.24 | 0.813 |
|  | Yes | 218 (20.07) | 1.29 | 3.37 | 0.001 |
|  | Ø | 1 (0.09) | Ø | Ø | Ø |
| Using curtain treatment | No | 1070 (98.53) | 1.12 | 0.62 | 0.533 |
|  | Yes | 15 (1.38) | 0.83 | -0.65 | 0.514 |
|  | Ø | 1 (0.09) | Ø | Ø | Ø |
| Using screens on windows | No | 931 (85.73) | 1.13 | 0.68 | 0.498 |
|  | Yes | 154 (14.18) | 0.92 | -0.93 | 0.355 |
|  | Ø | 1 (0.09) | Ø | Ø | Ø |
| Covering containers | No | 936 (86.19) | 1.11 | 0.57 | 0.566 |
|  | Yes | 149 (13.72) | 1.03 | 0.38 | 0.702 |
|  | Ø | 1 (0.09) | Ø | Ø | Ø |
| Emptying containers | No | 642 (59.12) | 1.22 | 0.95 | 0.340 |
|  | Yes | 443 (40.79) | 0.78 | -3.57 | < 0.001 |
|  | Ø | 1 (0.09) | Ø | Ø | Ø |
| Washing containers | No | 608 (55.99) | 1.16 | 0.76 | 0.450 |
|  | Yes | 477 (43.92) | 0.91 | -1.54 | 0.123 |
|  | Ø | 1 (0.09) | Ø | Ø | Ø |
| Using waste management | No | 876 (80.66) | 1.13 | 0.66 | 0.507 |
|  | Yes | 209 (19.24) | 0.93 | -1.11 | 0.266 |
|  | Ø | 1 (0.09) | Ø | Ø | Ø |
| Killing insects | No | 991 (91.25) | 1.13 | 0.67 | 0.504 |
|  | Yes | 94 (8.66) | 0.88 | -1.38 | 0.169 |
|  | Ø | 1 (0.09) | Ø | Ø | Ø |
| Using protective clothing | No | 1045 (96.22) | 1.13 | 0.67 | 0.506 |
|  | Yes | 40 (3.68) | 0.69 | -2.53 | 0.011 |
|  | Ø | 1 (0.09) | Ø | Ø | Ø |
| Using ventilator | No | 723 (66.57) | 1.41 | 2.07 | 0.039 |
|  | Yes | 28 (2.58) | 0.80 | -1.44 | 0.150 |
|  | Ø | 335 (30.85) | Ø | Ø | Ø |
| Using other means to protect against mosquitoes | No | 944 (86.92) | 1.10 | 0.54 | 0.588 |
|  | Yes | 141 (12.98) | 1.11 | 1.52 | 0.128 |
|  | Ø | 1 (0.09) | Ø | Ø | Ø |
| Using screens | No | 840 (77.35) | 1.14 | 0.76 | 0.447 |
|  | Yes | 229 (21.09) | 0.90 | -1.37 | 0.170 |
|  | Ø | 17 (1.57) | Ø | Ø | Ø |
| Bathroom is inside | No | 66 (6.08) | 1.33 | 1.39 | 0.165 |
|  | Yes | 1018 (93.74) | 0.83 | -1.84 | 0.066 |
|  | Ø | 2 (0.18) | Ø | Ø | Ø |
| Bathroom is outside | No | 996 (91.71) | 1.10 | 0.53 | 0.598 |
|  | Yes | 88 (8.1) | 1.16 | 1.75 | 0.080 |
|  | Ø | 2 (0.18) | Ø | Ø | Ø |
| Presence of entry points for mosquitoes into household | No | 786 (72.38) | 1.03 | 0.15 | 0.884 |
|  | Yes | 291 (26.8) | 1.34 | 4.42 | < 0.001 |
|  | Ø | 9 (0.83) | Ø | Ø | Ø |
| Presence of green areas near household | No | 550 (50.64) | 1.03 | 0.15 | 0.879 |
|  | Yes | 503 (46.32) | 1.16 | 2.48 | 0.013 |
|  | Ø | 33 (3.04) | Ø | Ø | Ø |
| Presence of water body near household | No | 317 (29.19) | 1.10 | 1.16 | 0.246 |
|  | Yes | 766 (70.53) | 1.08 | 0.39 | 0.700 |
|  | Ø | 3 (0.28) | Ø | Ø | Ø |
| Presence of vegetation inside | No | 308 (28.36) | 1.12 | 0.64 | 0.523 |
|  | Yes | 762 (70.17) | 0.99 | -0.10 | 0.920 |
|  | Ø | 16 (1.47) | Ø | Ø | Ø |
| Presence of vegetation in pots | No | 758 (69.8) | 1.13 | 0.68 | 0.498 |
|  | Yes | 304 (27.99) | 0.96 | -0.60 | 0.546 |
|  | Ø | 24 (2.21) | Ø | Ø | Ø |
| Presence of herb | No | 414 (38.12) | 1.33 | 2.04 | 0.041 |
|  | Yes | 408 (37.57) | 0.94 | -0.94 | 0.347 |
|  | Ø | 264 (24.31) | Ø | Ø | Ø |
| Presence of shrub | No | 370 (34.07) | 1.25 | 1.58 | 0.115 |
|  | Yes | 452 (41.62) | 1.08 | 1.20 | 0.230 |
|  | Ø | 264 (24.31) | Ø | Ø | Ø |
| Presence of vine | No | 809 (74.49) | 1.30 | 1.88 | 0.061 |
|  | Yes | 14 (1.29) | 1.04 | 0.23 | 0.816 |
|  | Ø | 263 (24.22) | Ø | Ø | Ø |
| Presence of garden | No | 572 (52.67) | 1.33 | 2.03 | 0.042 |
|  | Yes | 251 (23.11) | 0.92 | -1.11 | 0.269 |
|  | Ø | 263 (24.22) | Ø | Ø | Ø |
| Presence of tree | No | 478 (44.01) | 1.22 | 1.46 | 0.145 |
|  | Yes | 345 (31.77) | 1.16 | 2.35 | 0.019 |
|  | Ø | 263 (24.22) | Ø | Ø | Ø |
| Presence of other/decorative vegetation | No | 766 (70.53) | 1.27 | 1.72 | 0.086 |
|  | Yes | 57 (5.25) | 1.34 | 2.87 | 0.004 |
|  | Ø | 263 (24.22) | Ø | Ø | Ø |
| Presence of breeding sites | No | 817 (75.23) | 1.12 | 0.63 | 0.529 |
|  | Yes | 173 (15.93) | 1.21 | 2.91 | 0.004 |
|  | Ø | 96 (8.84) | Ø | Ø | Ø |
| Waste water disposal | Sewers | 692 (63.72) | 1.09 | 0.48 | 0.631 |
|  | Latrine | 8 (0.74) | 0.94 | -0.15 | 0.882 |
|  | Sceptic tank | 157 (14.46) | 1.09 | 0.48 | 0.631 |
|  | Other type | 225 (20.72) | 1.16 | 0.71 | 0.480 |
|  | Ø | 4 (0.37) | Ø | Ø | Ø |
| Waste collection | In a location inside household | 10 (0.92) | 0.53 | -1.42 | 0.156 |
|  | In a location outside household | 23 (2.12) | 2.46 | 2.04 | 0.042 |
|  | Private or municipal collection | 1047 (96.41) | 0.53 | -1.42 | 0.156 |
|  | Ø | 6 (0.55) | Ø | Ø | Ø |
| Waste disposal frequency | Daily | 678 (62.43) | 1.28 | 2.12 | 0.034 |
|  | Every other day | 377 (34.71) | 0.95 | -0.25 | 0.800 |
|  | Weekly | 10 (0.92) | 0.95 | -0.25 | 0.800 |
|  | Monthly | 1 (0.09) | 4.33 | 1.32 | 0.185 |
|  | Unpredictable | 17 (1.57) | 0.90 | -0.44 | 0.659 |
|  | Ø | 3 (0.28) | Ø | Ø | Ø |
